# Risk factors of late lesion growth after acute ischemic stroke treatment

**DOI:** 10.3389/fneur.2022.977608

**Published:** 2022-10-05

**Authors:** Praneeta Konduri, Amber Bucker, Anna Boers, Bruna Dutra, Noor Samuels, Kilian Treurniet, Olvert Berkhemer, Albert Yoo, Wim van Zwam, Robert van Oostenbrugge, Aad van der Lugt, Diederik Dippel, Yvo Roos, Joost Bot, Charles Majoie, Henk Marquering, the MR CLEAN Trial Investigators (Multicenter Randomized Clinical Trial of Endovascular Treatment for Acute Ischemic Stroke in the Netherlands)

**Affiliations:** ^1^Department of Biomedical Engineering and Physics, Amsterdam UMC, Location AMC, Amsterdam, Netherlands; ^2^Department of Radiology and Nuclear Medicine, Amsterdam UMC, Location AMC, Amsterdam, Netherlands; ^3^Department of Radiology, University Medical Center Groningen, Groningen, Netherlands; ^4^Nico-Lab, Amsterdam, Netherlands; ^5^Department of Radiology and Nuclear Medicine, Erasmus MC, University Medical Center, Rotterdam, Netherlands; ^6^Department of Neurology, Erasmus MC University Medical Center, Rotterdam, Netherlands; ^7^Department of Public Health, Erasmus MC, University Medical Center, Rotterdam, Netherlands; ^8^Department of Radiology, Haaglanden Medisch Centrum, The Hague, Netherlands; ^9^Department of Radiology, Texas Stroke Institute, Dallas-Fort Worth, Dallas, TX, United States; ^10^Department of Radiology and Nuclear Medicine, Maastricht University Medical Center and Cardiovascular Research Institute Maastricht (CARIM), Maastricht, Netherlands; ^11^Department of Neurology, Maastricht University Medical Center and Cardiovascular Research Institute Maastricht (CARIM), Maastricht, Netherlands; ^12^Department of Neurology, Amsterdam UMC, Location AMC, Amsterdam, Netherlands; ^13^Department of Radiology and Nuclear Medicine, Amsterdam UMC, Vrije Universiteit van Amsterdam, Amsterdam, Netherlands

**Keywords:** risk factors, predictors, post-treatment, subacute, lesion evolution, infarct growth, acute ischemic stroke

## Abstract

**Background:**

Even days after treatment of acute ischemic stroke due to a large vessel occlusion, the infarct lesion continues to grow. This late, subacute growth is associated with unfavorable functional outcome. In this study, we aim to identify patient characteristics that are risk factors of late, subacute lesion growth.

**Methods:**

Patients from the MR CLEAN trial cohort with good quality 24 h and 1-week follow up non-contrast CT scans were included. Late Lesion growth was defined as the difference between the ischemic lesion volume assessed after 1-week and 24-h. To identify risk factors, patient characteristics associated with lesion growth (categorized in quartiles) in univariable ordinal analysis (*p* < 0.1) were included in a multivariable ordinal regression model.

**Results:**

In the 226 patients that were included, the median lesion growth was 22 (IQR 10–45) ml. In the multivariable model, lower collateral capacity [aOR: 0.62 (95% CI: 0.44–0.87); *p* = 0.01], longer time to treatment [aOR: 1.04 (1–1.08); *p* = 0.04], unsuccessful recanalization [aOR: 0.57 (95% CI: 0.34–0.97); *p* = 0.04], and larger midline shift [aOR: 1.18 (95% CI: 1.02–1.36); *p* = 0.02] were associated with late lesion growth.

**Conclusion:**

Late, subacute, lesion growth occurring between 1 day and 1 week after ischemic stroke treatment is influenced by lower collateral capacity, longer time to treatment, unsuccessful recanalization, and larger midline shift. Notably, these risk factors are similar to the risk factors of acute lesion growth, suggesting that understanding and minimizing the effects of the predictors for late lesion growth could be beneficial to mitigate the effects of ischemia.

## Introduction

Treatment of acute ischemic stroke (AIS) is strongly focused on the acute phase. However, even after treatment, both successful and unsuccessful, the volumetric increase of infarct related lesions, occurs between 24 h and 1 week after treatment. Although commonly unrecognized, this late lesion growth in the subacute period (i.e., after 24 h of stroke onset) is associated with unfavorable functional outcome (1–6). This suggests that secondary treatment even 24 h after stroke onset can be beneficial to alleviate the effects of ischemia. Infarct-related lesion growth is caused by a combination of edema formation and true infarct progression. Lesion growth is a dynamic and often an irreversible process (7, 8). Both ischemia and post-ischemic reperfusion lead to a cascade of pathophysiological processes, which results in poor prognosis (9). Although several risk factors for early, acute lesion growth between pre and post treatment time-points have been identified in literature (10–13). risk factors for late lesion growth after treatment are understudied.

The aim of this study is to perform an exploratory analysis of associations between baseline, imaging, and (post-) treatment characteristics and late lesion growth after treatment in patients with acute ischemic stroke due to a large vessel occlusion.

## Methods

### Patient population

For this study, we included data from patients included in the Multicenter Randomized Clinical Trial of Endovascular Treatment for Acute Ischemic Stroke in the Netherlands (MR CLEAN). A comprehensive description of the methodology of the MR CLEAN trial has been reported previously (14). In the MR CLEAN trial, patients with AIS due to a proximal arterial occlusion in the anterior circulation, aged 18 years or older with the possibility to treat with endovascular thrombectomy (EVT) within 6 h of symptom onset were randomized between standard with intravenous treatment (IVT) or standard care with IVT followed by EVT. Patients (allocated to intervention or control group) with Non-Contrast Computed Tomography (NCCT) scans between 24 h and 7 days after stroke onset were included. Patients whose scans had movement artifacts, partial volume effects and other technical errors were excluded. Further details of the inclusion and exclusion criterion of this study are provided in the online Supplemental Material Figure 1 (1). The study protocol was approved by a central medical ethics committee and the research board of each participating center. All included patients or their legal representatives provided written informed consent.

### Image analysis

In this study, lesion was defined as recent (i.e., early subacute) ischemic tissue as visualized on NCCT. Lesion was delineated to include infarcted tissue, hemorrhage and edema as described in a previous study (1). In short, the segmentations on 24-h NCCT scan were manually delineated using ITK-SNAP by two trained observers (A.B and P.K) (15). To prevent disparities while assessing the lesion, a fixed window-level setting with a window width 30 HU and center level 35 HU was used. Hyper-densities next to or within the hypodense areas including edema extending into the contra-lateral hemisphere or causing ventricular and/or sulcal effacement were considered to be part of the lesion. The observers were informed about the affected hemisphere. Old ipsilateral lesions, which were more hypodense with distinct borders and/or with no mass effect were not included in the segmentation. Lesion volume after 1 week (5–7 days) of stroke onset was automatically identified on NCCT scans using a previously validated software (16). The segmentations were verified and adjusted if necessary by one of two experienced neuro-radiologists (C.B.L.M. and J.C.J.B.) who were blinded to baseline and outcome characteristics. The lesions are more discernible on the 1-week NCCT scans compared to the 24-h NCCT scans and our software performed better in segmenting the lesions on the 1-week. Hence, we used the software for identifying lesions on the 1-week scans and manually delineated the 24-h lesions, often presented as subtle changes on the 24-h scans.

The lesion volume was calculated by multiplying the number of voxels in the segmentation with the image voxel size. Late lesion growth was the difference between lesions volumes calculated after 1 week and 24 h.

Midline shift, known as the gold standard to assess the extent of edema, was defined as the horizontal displacement (in mm) of midline structures and was measured by a neuroradiologist at the level of the septum pellucidum as described in a previous study (17). A central blinded core lab scored other radiological parameters like the Alberta Stroke Program Early Computed Tomography Score (ASPECTS) (18), Collateral Score (CS) (19), and modified Arterial Occlusion Lesion (mAOL) score (20). In this study, successful recanalization was assessed on the 24-h follow up CT Angiography scan and was defined as mAOL score of 3 points.

### Statistical analysis

Visual inspection of the histogram of late lesion growth before and after log transformation and the Shapiro Wilk test indicated that it was not normally distributed (Supplemental material Figure II). Given the proof-of-concept and exploratory nature of this study, we modeled the late lesion growth by dividing the variable into quartiles instead of modeling it as a continuous variable for comprehensibility. Baseline, imaging and (post) treatment characteristics of the study population are tabulated according to quartiles of late lesion growth. Continuous variables are expressed as or medians (interquartile ranges, IQR), where applicable. Categorical variables are expressed as numbers of patients and percentages.

To identify risk factors for late lesion growth, we performed ordinal logistic regression, which is also known as shift analysis. The resulting common odds ratios represent a shift toward the higher quartile of late lesion growth after assuming proportional odds between different quartiles. To understand the effect of different variables we first performed univariable ordinal regression with baseline, image, and (post-) treatment characteristics with late lesion growth (as a categorial variable). A multivariable ordinal regression was performed including all variables that were associated with late lesion growth in the univariable analysis with a *p*-value of < 0.10. To visualize the association of the risk factors with late lesion growth identified in the multivariable ordinal regression, this relation was plotted while setting the other variables in the multivariable model to their mean values (21).

Patients with missing information were excluded from the analysis. Statistical analyses were performed using SPSS (IBM SPSS Statistics, version 26, 2019) and R [Version 4.0.2 (2020-06-22)] using RStudio (Version 1.2.5033–© 2009-2019 RStudio, Inc.). A *p*-value ≤ 0.05 was considered statistically significant.

## Results

### Patient characteristics

A total of 226 patients met the inclusion criterion for this study (Supplemental material Figure I). The median age of the population was 67 (IQR: 57–76) years and 132 (58%) were male. Nineteen (8.4%) patients suffered from a previous ischemic stroke, and 67 (30%) patients were using statins. Baseline median NIHSS was 17 (IQR: 13–21) and 63 (28%) patients had a proximal occlusion in the Intracranial Carotid Artery (ICA) or the ICA-terminus, while 163 patients had a distal occlusion in the middle cerebral artery [M1 segment: 144 (64%), M2 segment: 18 (8%)] and anterior cerebral artery (A2 segment: 1). Two-hundred-four (90%) patients received intra-venous thrombolysis, 106 (47%) were allocated to endovascular treatment, and 110 (54%) patients in our study population achieved successful recanalization. Furthermore, of the patients allocated to EVT, 60 patients (57%) achieved a modified thrombolysis in cerebral infarction score of 2b-3 as assessed on the procedural digital subtraction angiography scans. Patient characteristics of the population are provided in Table 1 and patient characteristics categorized in quartiles of late infarct growth are provided in the (Supplementary Material Table I).

**Table 1:** Baseline, radiological and treatment characteristics of the study population and univariable ordinal regression of these characteristics with subacute lesion evolution.

**Variable**	**Population**	**Common odds ratio (95% CI)**	***p*-value**
Age	67 (57–76)	1.00 (0.98–1.02)	0.96
Sex	132 (58%)	0.94 (0.59–1.51)	0.81
**Previous medical history**
Previous ischemic stroke	19 (8.4%)	1.3 (0.53–3.20)	0.57
Myocardial infarction	27 (12%)	0.71 (0.35–1.42)	0.33
Diabetes mellitus	25 (11%)	1.50 (0.71–3.20)	0.29
Hypertension	113 (50%)	0.96 (0.60–1.53)	0.86
Atrial fibrillation	64 (28%)	1.23 (0.73–2.07)	0.43
Hypercholesterolemia	54 (24%)	0.93 (0.54–1.59)	0.78
Current smoking	66 (29%)	0.76 (0.46–1.27)	0.30
**Previous medication**
Antiplatelet drugs	63 (28%)	0.77 (0.46–1.29)	0.32
Coumarins	15 (6.6%)	1.18 (0.47–2.98)	0.72
Statins	67 (30%)	0.92 (0.56–1.52)	0.74
Anti-hypertensive drugs	114 (50%)	1.06 (0.66–1.69)	0.81
**Clinical parameters**
Pre-stroke modified Rankin Scale (0–2)	216 (96%)	1.00 (0.31–3.25)	1.00
Systolic blood pressure (mmHg)	140 (130–160)	1.01 (1.00–1.02)	0.04^*^
Clinical hemisphere side left	123 (54%)	0.93 (0.58–1.49)	0.77
Baseline NIHSS	17 (13–21)	1.07 (1.03–1.12)	<0.01^**^
**Radiological parameters**
ASPECT score	9 (8–10); 2 (0.88%)	0.81 (0.70–0.94)	0.01^**^
Proximal occlusion (ICA or ICA-T)	63 (28%)	1.76 (1.03–3.00)	0.04^*^
Collateral score	2 (1–3); 2 (0.88%)	0.61 (0.46–0.82)	<0.01^**^
**Treatment characteristics**
Received iv Thrombolysis	204 (90%)	0.92 (0.41–2.07)	0.84
Allocated to endovascular treatment	106 (47%)	0.77 (0.48–1.24)	0.28
Time to randomization	200 (150–260)	1.04 (1.01–1.08)	0.01^**^
**24-h follow up characteristics**
Successful recanalization	110 (54%); 23 (10%)	0.59 (0.36–0.97)	0.04^*^
Lesion volume (ml)	43 (21–99)	1.01 (1.01–1.02)	<0.01^**^
Midline shift (mm)	0 (0–2.8)	1.27 (1.12–1.44)	<0.01^**^

All data in the “Population” column are displayed as median (interquartile range) or number (percentage of population). When required number of missing patients (percentage of population) is also provided in the “Population” column. All variables significant at the level of p < 0.10 have been selected in the multivariable regression model. ASPECTS, Alberta Stroke Program Early Computed Tomography Score; ICA, Intracranial carotid artery; ICA-T, Intracranial carotid artery-T junction; NIHSS, NIH Stroke Scale/Score.

** p ≤ 0.01

* p ≤ 0.05.

### Lesion characteristics

**Figure 1:**
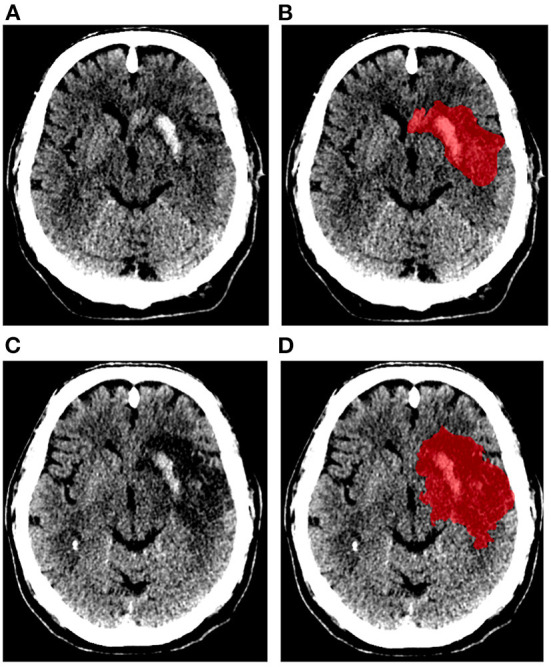
Example of ischemic lesion segmentation (represented in red). Non-contrast CT scans and segmentation of ischemic lesion obtained after 24 h **(A,B)**, and 1 week **(C,D)** of stroke onset.

Figure 1 shows an example of the delineation of the lesions on the 24-h and 1-week NCCT scans. The median lesion volume after 24 h was 43 (IQR: 21–99) ml and after 1 week was 79 (IQR: 33–140) ml. Median midline shift measured on the 24-h NCCT scans was 0 (IQR: 0–2.8) mm and 140 (62%) patients had no midline shift. The median late lesion growth was 22 (IQR: 10–45) ml. Patients were categorized into minimal growth (up to 10 ml), low growth (10–22 ml), moderate growth (22–45 ml), and large growth (>45 ml). The median lesion growth for the categories were: minimal 1.6 (IQR −2.9 to 6.3) ml; low 16 (IQR: 14–19) ml; moderate 32 (IQR: 26–36) ml; and large 73 (IQR: 60–95) ml, respectively.

### Regression analysis

Results of the univariable ordinal regression analysis are provided in Table 1. In summary, pre-treatment characteristics such as higher systolic blood pressure (*p* = 0.04), higher NIHSS (*p* < 0.01), lower ASPECTS (*p* = 0.01), lower collateral score (*p* < 0.01), longer time to randomization (*p* = 0.01) were associated with lesion growth in the univariable analyses. Furthermore, unsuccessful recanalization (*p* = 0.04), larger 24-h lesion volume (*p* < 0.01) and 24-h midline shift (*p* < 0.01) were associated with late lesion growth. Administering intravenous thrombolysis (*p* = 0.84) and allocation to endovascular treatment (*p* = 0.28) were not associated with late lesion growth.

In multivariable ordinal regression analysis, lower collateral score [aOR: 0.62 (0.44–0.87); *p* = 0.01], longer time to treatment [aOR: 1.04 (1.00–1.08); *p* = 0.04], unsuccessful recanalization [aOR: 0.57 (0.34–0.97); *p* = 0.01] and larger 24-h midline shift [aOR: 1.18 (1.02–1.36); *p* = 0.02] were independently associated with late lesion growth. Higher NIHSS on hospital admission [aOR: 1.05 (1.00–1.10); *p* = 0.06] and presence of a proximal occlusion [aOR: 1.70 (0.94–3.1); *p* = 0.08] showed a non-significant trend to be associated with late lesion growth (Table 2). The association of these variables on the predicted probability of the late lesion growth category is visualized in the (Supplementary Material Figure III).

**Table 2:** Multivariable ordinal regression of subacute lesion evolution modeled in quartiles with baseline, imaging, and post-treatment characteristics.

**Variable**	**Adjusted common odds ratio (95% CI)**	***p*-value**
Systolic blood pressure (mmHg)	1.00 (0.99–1.01)	0.57
Baseline NIHSS	1.05 (1.00–1.10)	0.06^†^
ASPECT score	1.00 (0.83–1.20)	0.99
Proximal occlusion (ICA or ICA-T)	1.70 (0.94–3.1)	0.08^†^
Baseline collateral score	0.62 (0.44–0.87)	0.01^*^
Time to randomization (per 10 min)	1.04 (1.00–1.08)	0.04^*^
Successful recanalization	0.57 (0.34–0.97)	0.04^*^
Midline shift (mm)	1.18 (1.02–1.36)	0.02^*^

ASPECTS, Alberta Stroke Program Early Computed Tomography Score; ICA, Intracranial carotid artery; ICA-T, Intracranial carotid artery-T junction; NIHSS, NIH Stroke Scale/Score.

* p ≤ 0.05

† p<0.1.

## Discussion

In this exploratory study, we identified lower collateral score, longer time between onset and treatment, unsuccessful recanalization, and larger midline shift to be risk factors of subacute late lesion growth after treatment.

In literature, risk factors have been identified for lesion growth expansion between baseline and follow up at acute (6–24 h) (10, 12, 22), subacute (24 h to 7 days) (11, 23–29), or chronic (more than 7 days) (13) time points. In these studies, lesion growth was assessed on different modalities using various thresholds. Nevertheless, it is interesting to note that in line with our findings, unsuccessful reperfusion was associated with lesion growth between baseline and follow-up at acute (10, 12, 22) and subacute (11, 26) time points by several other studies, except by Haussen et al. (29). Our finding of lack of reperfusion to be a risk factor for late lesion growth in the subacute period is consistent with the results of previous studies that assessed lesion growth in the subacute period on MRI (4). It is well acknowledged that successful restoration of blood flow salvages the at-risk tissue in the down-stream territory and limits post-treatment lesion expansion in the acute situation. But, lesion progression continues possibly due to thrombus fragmentation to new or to the downstream territories, formation of micro-emboli or damage to the capillaries and smaller vasculature that could result from incomplete reperfusion, especially in cases with unsuccessful recanalization (12, 29). In contrast, Sah et al. did not find similar influence of reperfusion on lesion growth between 5 and 24 h after treatment, probably owing to their small sample size (5). In our study, successful recanalization is assessed in patients that were and in the patients that were not allocated to receive EVT. Since the recanalization rates are higher in the patients allocated to receive EVT (74%) compared to those that did not receive EVT (27%), it is possible that our observations may be biased to the control arm of the trial. Nevertheless, unsuccessful recanalization, irrespective of treatment arm, is associated with late lesion growth and treatment allocation was not. In this exploratory study we aimed to identify potential risk factors of late lesion growth and not to build an optimal association or prediction model. We, therefore, did not account for possible collinearities between the independent variables in the multivariable analysis.

In our study, we found that lower baseline collateral capacity is a risk factor for late lesion growth. Man et al. also identified low collateral supply to be associated with lesion growth between baseline and follow-up at a sub-acute time point (11). However, in a subpopulation of the DEFUSE3 patients that received an unscheduled MRI or CT scan after 5 days, Tate et al. found a statistically insignificant trend between good collateral circulation and larger lesion growth in the subacute period (30). Consistent with Campbell et al., they suggest that a better baseline collateral supply leads to a smaller lesion in the early time window, which can expand if the collaterals fail in the later time window, especially in patients with unsuccessful reperfusion (23). This discrepancy in findings could be because Tate et al. compared lesions measured mainly on MRI after 24 h and NCCT after 5 days, unlike in our study where we used NCCT at both time points. This could also explain the lower median lesion growth compared to that observed in our study. Moreover, in the MR CLEAN trial, obtaining NCCT after 24 h and 1 week was standard practice unlike the DEFUSE 3 trial, which performed an unscheduled scan at 5 days only in the patients with a worse outcome. Studying collateral circulation in the subacute period would help to better understand the influence of collaterals on late lesion growth (14, 30).

Some of the (plausible) risk factors of the late lesion growth identified in this study, like longer time to randomization, poorer collaterals, higher baseline NIHSS, presence of a proximal occlusion are known to also be associated with larger lesion growth between baseline and acute time points, leading to larger 24-h lesion volumes (10). Furthermore, recanalization status was assessed on the follow-up CT Angiography scans, which is generally acquired after 24 h. Including time to randomization (as a proxy for time to treatment) instead of time to recanalization as an independent variable in the regression models captures the variability of time to treatment and its influence on late lesion growth better in our population. It is likely that influence of the risk factors on late lesion growth is due to the associated 24-h ischemic lesion volumes and the ensuing deterioration. But, incorporating the 24-h lesion volume in the multivariable model could obscure the influence of the other risk factors on the late lesion growth, especially because of the correlation (collinearity) between these risk factors and the 24-h lesion volume. Simard et al. have found that ischemia leads to the deterioration of the blood brain barrier, loss of capillary integrity and development of edema which leads to swelling of tissue. Swelling increases tissue pressure and exerts further mechanical force on the adjacent tissues, thereby reducing capillary flow. This further foster infarct growth and development of edema. The effect of edema is most pronounced between 24 and 72 h after an ischemic event (9). Presence and extent of midline shift is an indicator for mass effect. In our study, we use midline shift as a proxy for the 24-h lesion volume in the multivariable model to identify risk factors of late lesion growth. Midline shift is a surrogate marker for edema, especially for lesions large enough to cause a displacement in the midline structures (17). Our observation that increased midline shift is associated with late lesion growth is most likely due to the prominent effect of a large 24-h lesion volume and the subsequent deterioration of the blood brain barrier and loss of capillary integrity, which also promotes the formation of vasogenic edema.

Our study has some limitations. In our study, we use NCCT imaging to assess the affected tissue 24 h and 1 week after stroke. It is important to note that lesions identified on NCCT scans include infarcted tissue, edema (cytotoxic and/or vasogenic) and even hemorrhagic regions. Furthermore, they are rather subtle on the 24-h scans further complicating the delineation of the lesions. Nevertheless, a recent study showed that the lesion is discernible in 89% of the patients on the 24-h NCCT scan (31). Still, NCCT was used in the trial since NCCT is a more commonly available imaging modality in most hospitals, especially when multiple scans are acquired at different timepoints. Furthermore, use of MRI imaging can lead to loss of inclusions because of contraindications to MRI and patients' uncooperativeness (possibly due to claustrophobia) to undergo an MRI. Since the effect of edema is maximal in the subacute period after an ischemic event (1), future studies focused on identifying risk factors for progression of the lesion after distinguishing edema and lesion are necessary to gain a better insight into the pathophysiology of lesion progression. DWI and MR FLAIR are better imaging techniques for assessing lesion status and its constituents especially in the early subacute window (after 24 h) compared to CT. Hence, we advocate that studying late lesion growth assessed on MRI would also aid in better understanding this phenomenon. The 1-week follow-up NCCT scan was obtained 3–9 days after stroke onset, which further necessitates the identification of edema and true infarcted regions within the lesions (14). It is known that reperfusion is a more commonly used marker for treatment success compared to recanalization. Since reperfusion is generally assessed on digital subtraction angiography images that are only available for patients in the intervention arm, in this study, treatment success was determined on the 24-h follow up CT angiography scan. Further studies distinguishing lesion progression into downstream and new territories after considering the influence of reperfusion are also required.

## Conclusion

Lesion growth in patients treated for an AIS is not restricted to the acute phase between stroke onset and (successful) reperfusion. We identified lower collateral capacity, longer time between stroke onset and treatment, unsuccessful recanalization, and larger midline shift as risk factors for late, subacute lesion growth. Furthermore, higher baseline NIHSS and presence of a proximal occlusion showed a trend to be associated with more subacute lesion evolution. Further research should be directed toward understanding and minimizing the effects of the predictors for late lesion growth.

## Data availability statement

The data analyzed in this study was obtained from the Multicenter Randomized Clinical Trial of Endovascular Treatment for Acute Ischemic Stroke in the Netherlands (MR CLEAN; https://www.mrclean-trial.org/home.html), the following licenses/restrictions apply: Due to the sensitive nature of the data these datasets are not readily available. Requests to access these datasets should be directed to the MR CLEAN executive committee, mrclean@erasmusmc.nl.

## Ethics statement

The studies involving human participants were reviewed and approved by MR CLEAN trial Committee. The patients/participants provided their written informed consent to participate in this study.

## Author contributions

WZ, AL, RO, DD, YR, and CM: designed the MR CLEAN trial. OB: collected and prepared the data for the trial. PK, OB, BD, ABu, ABo, JB, and CM: prepared data for this study. PK: performed the statistical analysis, interpreted the results, and drafted the paper. HM: assisted with the statistical analysis, interpretation of the results, and drafting the paper. NS, ABu, KT, OB, AY, WZ, RO, AL, DD, YR, JB, and CM: critically revised the paper. All authors contributed to the article and approved the submitted version.

## Funding

MR CLEAN is partly funded by the Dutch Heart Foundation (2008 T030). MR CLEAN is also funded by unrestricted grants from: AngioCare BV, Covidien/EV3^®^, MEDAC Gmbh/LAMEPRO, Penumbra Inc., and Concentric Medical/TOP Medical BV. The study is designed, conducted, analyzed, and interpreted by the investigators independently of all. This sub-study is also funded by INSIST (www.insist-h2020.eu): a European Union's Horizon 2020 research and innovation program (grant agreement number: 777072).

## Conflict of interest

PK is funded by INSIST (www.insist-h2020.eu): a European Union's Horizon 2020 research and innovation program (grant agreement number: 777072). ABo is a shareholder of Nico.Lab. AY reports grants from Cerenovus Neurovascular, Medtronic, Stryker, Penumbra, and Genentech for investigator-initiated studies; funds from Stryker, Cerenovus Neurovascular and Penumbra (core imaging lab activities) and Genentech (consultation); and declares to have equity ownership from Insera Therapeutics. WZ reports speaker fees from Stryker and Cerenovus (paid to the institution). AL and DD report funds from the Cerenovus Neurovascular, Dutch Heart Foundation, Brain Foundation Netherlands, Organization for Health Research and Development, Health Holland Top Sector Life Sciences & Health, and unrestricted grants paid to the institution from AngioCare BV, Covidien/EV3, MEDAC Gmbh/LAMEPRO, PenumbraInc., Top Medical/Concentric, Stryker, Stryker European Operations BV, Medtronic, Thrombolytic Science and LLC for research. AL further reports grants paid to the institution from the Siemens Healthineers, GE Healthcare and Philips Healthcare. YR is a shareholder at Nico-Lab. CM reports grants from European Commission, during the conduct of the study; grants from CVON/Dutch Heart Foundation, grants from TWIN Foundation, grants from Stryker, outside the submitted work; and owns stock in Nico.lab. HM is a Co-founder and shareholder of Nico.lab. The remaining authors declare that the research was conducted in the absence of any commercial or financial relationships that could be construed as a potential conflict of interest.

## Publisher's note

All claims expressed in this article are solely those of the authors and do not necessarily represent those of their affiliated organizations, or those of the publisher, the editors and the reviewers. Any product that may be evaluated in this article, or claim that may be made by its manufacturer, is not guaranteed or endorsed by the publisher.

## Abbreviations

aOR: adjusted odds ratio
ASPECTS: Alberta Stroke Program Early Computed Tomography Score
CT: computed tomography
CI: confidence interval
EVT: endovascular treatment
IQR: inter quartile range
mAOL: modified Arterial Occlusion Lesion
mRS: modified Rankin Scale
MR CLEAN: Multicenter Randomized Clinical Trial of Endovascular Treatment for Acute Ischemic Stroke in the Netherlands
NIHSS: NIS Stroke Scale
NCCT: non-contrast CT
OR: odds ratio.
