# Supplementary material for: Risk factors of late lesion growth after acute ischemic stroke treatment
Source: Front Neurol. 2022 Oct 5;13:977608. doi: 10.3389/fneur.2022.977608 (PMC9581245; doi:10.3389/fneur.2022.977608)
Supplement: Supplementary file 1 [file Table_1.pdf]

Supplementary Material

1 Tables

Table I: Comparison of baseline, clinical and treatment characteristics between the quartiles of subacute lesion evolution for 226 patients

Variables	Population	Sub-acute lesion evolution				p-value
		<10 ml	<22 ml	<45 ml	≥45 ml	
Age	67(57-76)	66(52-77)	67(59-76)	66(57-76)	67(57-76)	0.99
Sex	132(58%)	35(61%)	29(52%)	37(66%)	31(54%)	0.40
**Previous medical history**						
Previous ischemic stroke	19(8.4%)	6(11%)	2(3.6%)	4(7.1%)	7(12%)	0.35
Myocardial infarction	27(12%)	7(12%)	9(16%)	7(13%)	4(7.0%)	0.52
Diabetes mellitus	25(11%)	6(11%)	3(5.4%)	8 (14%)	8(14%)	0.40
Hypertension	113(50%)	33 (58%)	23(41%)	26(46%)	31(54%)	0.27
Atrial fibrillation	64(28%)	15 (26%)	14(25%)	17(30%)	18(32%)	0.84
Hypercholesterolemia	54(24%)	14(25%)	11(20%)	19 (34%)	10(18%)	0.18
Current smoking	66(29%)	19(33%)	17(30%)	16(29%)	14(25%)	0.77
**Previous medication**						
Antiplatelet drugs	63(28%)	18(32%)	15(27%)	18(32%)	12(21%)	0.52
Coumarins	15(6.6%)	3(5.3%)	4(7.1%)	4(7.1%)	4(7.0%)	0.97
Statins	67(30%)	18(32%)	12(21%)	25(45%)	12(21%)	0.02*
Anti-hypertensive drugs	114(50%)	30(53%)	25(45%)	29(52%)	30(53%)	0.80
**Clinical parameters**						
Pre-stroke modified Rankin Scale (0-2)	216(96%)	54(95%)	54(96%)	54(96%)	54(95%)	0.94
Systolic blood pressure (mmHg)	141 (130-160)	140 (125-160)	140 (130-156)	144 (127-160)	145 (130-165)	0.45
Clinical hemisphere side left	123(54%)	30(53%)	33(59%)	31(55%)	29 (51%)	0.84
Baseline NIHSS	17(13-21)	16(11-20)	16(14-21)	18(14-21)	19(16-22)	0.03*
**Baseline radiological parameters**						
ASPECT score<br>Missing	9(8-10)<br>2 (0.88%)	9(8-10)	9(8-10)	9(8-10)	8(7-10)	0.11
Proximal occlusion (ICA or ICA-T)	63(28%)	13 (23%)	13(23%)	14(25%)	23(40%)	0.11
Collateral score<br>Missing	2(1-3)<br>2(0.88%)	2(2-3)	2(2-3)	2 (1-3)	2 (1-2)	<0.01**
**Treatment characteristics**						
Received iv Treatment	204(90%)	51(90%)	51(91%)	52(93%)	50(88%)	0.82
Allocated to endovascular treatment	106(47%)	31(54%)	25(45%)	25(45 %)	25(44%)	0.63
Time to	200	190	210	190	230	0.27

randomization(minutes)	(150-260)	(140-240)	(160-250)	(160-250)	(150-280)	
**24-hour follow up characteristics**						
Successful recanalization	110(54%)	34(64%)	28(57%)	25(50%)	23(45%)	0.23
Missing	23 (10%)					
Lesion volume (ml)	43(21-99)	18(8.8-42)	27(19-55)	60(34-87)	83(44-160)	<0.01**
Midline shift (mm)	0(0-2.8)	0(0-0)	0(0-0.26)	0(0-2.8)	1.7(0-4.1)	<0.01**

All data are displayed as median (interquartile range) or number (percentage of population). Kruskal Wallis test and Chi-Square tests were performed to compare continuous and binary/categorical variables between the different quartiles of lesion evolution respectively. Abbreviations - ASPECTS: Alberta Stroke Program Early Computed Tomography Score ICA: Intracranial carotid artery ICA-T: Intracranial carotid artery-T junction; NIHSS: NIH Stroke Scale/Score ** $p \leq 0.01$ * $p \leq 0.05$

2 . Supplementary Figures

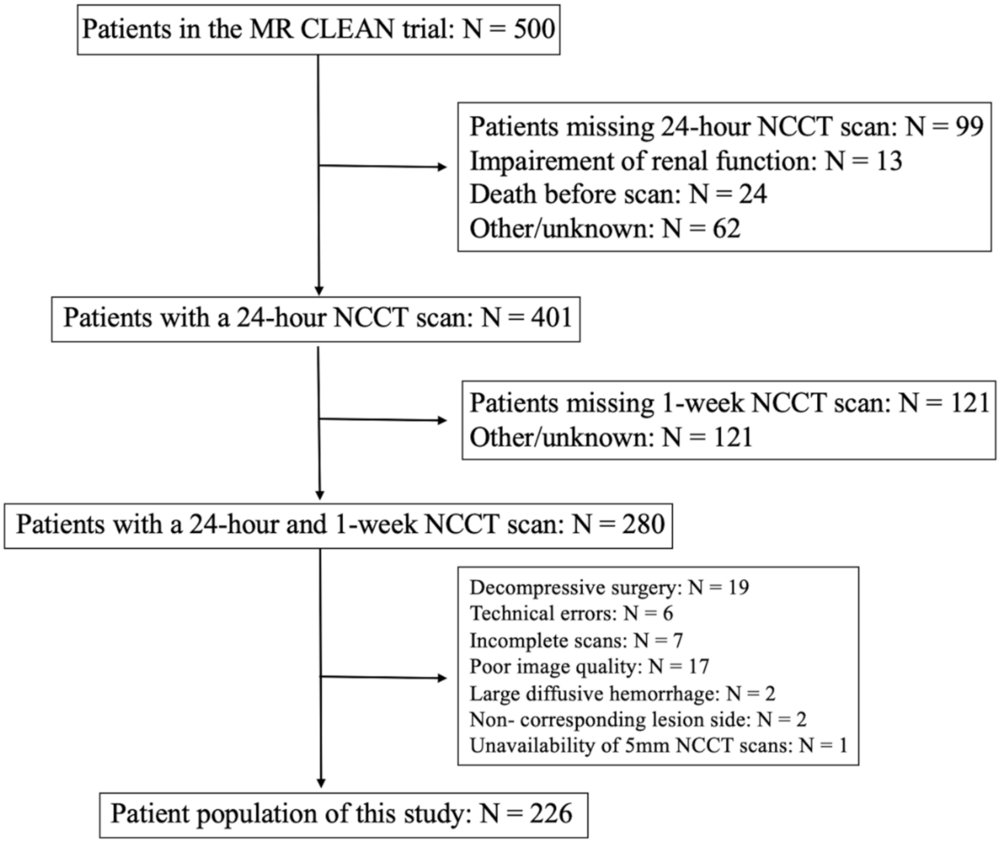

Supplementary Figure 1: Flowchart describing inclusion criterion of the study

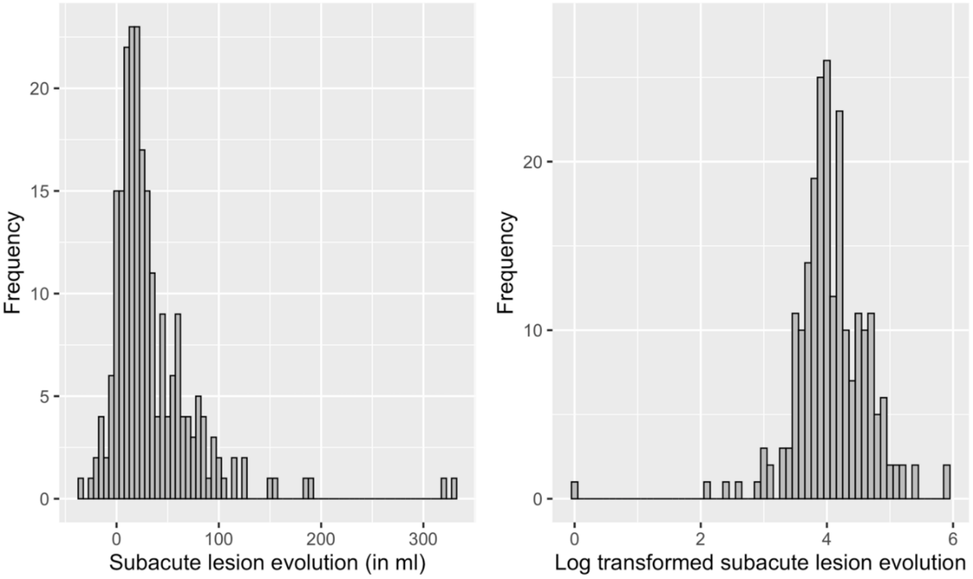

Supplementary Figure 2: Histogram of (left) subacute lesion evolution (in ml) and (right) log transformed lesion evolution to assess normality. The skewness and kurtosis values of lesion evolution were 3.2 and 19, respectively. Similarly, the skewness and kurtosis values of log transformed lesion evolution were -1.3 and 13, respectively.

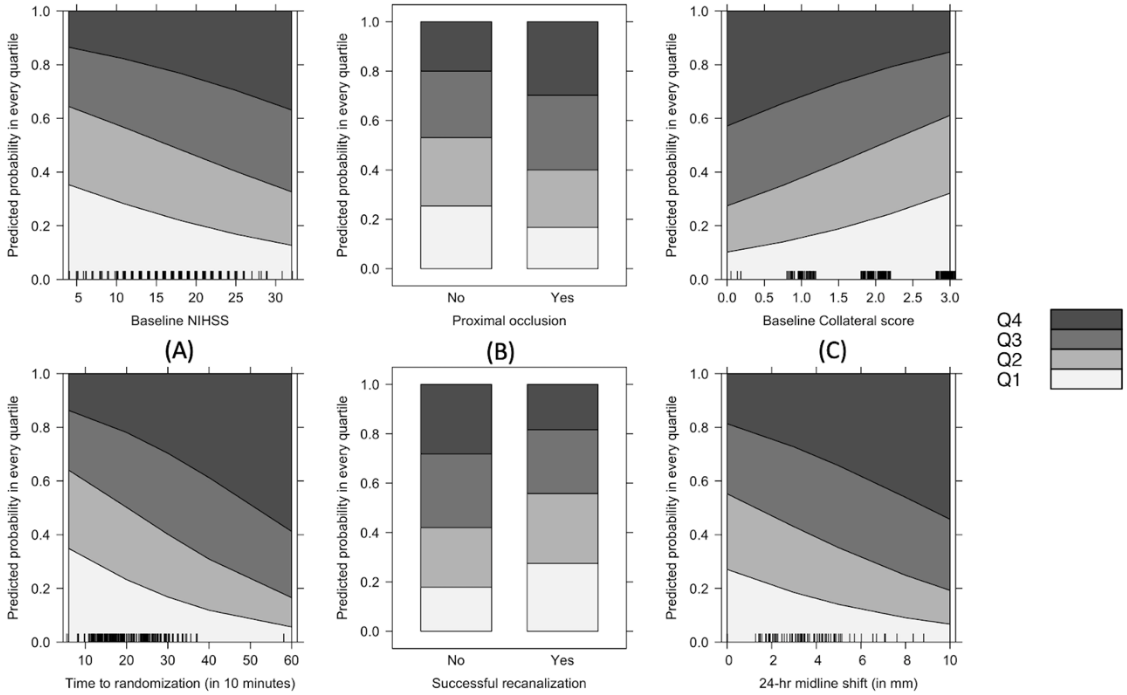

Supplementary Figure 3: Effect of (A) baseline NIH Stroke Scale, (B) proximal occlusion, (C) baseline collateral score, (D) time to randomization, (E) successful recanalization and (F) 24-hour midline shift on probability of each quartile of subacute lesion evolution

3 MR CLEAN trial investigators

Olvert A. Berkhemer, Amsterdam UMC, location AMC, the Netherlands and Erasmus MC-University Medical Center Rotterdam, the Netherlands. Puck S.S. Fransen, Erasmus MC-University Medical Center Rotterdam, the Netherlands. Debbie Beumer, Erasmus MC-University Medical Center Rotterdam, the Netherlands and Maastricht University Medical Center and Cardiovascular Research Institute Maastricht (CARIM), the Netherlands. Berkhemer, Fransen, and Beumer contributed equally. Lucie A. van den Berg, Amsterdam UMC, location AMC, the Netherlands. Hester F. Lingsma, Erasmus MC-University Medical Center Rotterdam, the Netherlands. Albert J. Yoo, Massachusetts General Hospital, Boston, United States of America. Wouter J. Schonewille, Saint Antonius Hospital, Nieuwegein, the Netherlands. Jan Albert Vos, MD, Sint Antonius Hospital, Nieuwegein, the Netherlands. Paul J. Nederkoorn, Amsterdam UMC, location AMC, the Netherlands. Marieke J.H. Wermer and Marianne A.A. van Walderveen, Leiden University Medical Center, the Netherlands. Julie Staals, Maastricht University Medical Center and Cardiovascular Research Institute Maastricht (CARIM), the Netherlands. Jeannette Hofmeijer and Jacques A. van Oostayen, Rijnstate Hospital, Arnhem, the Netherlands. Geert J. Lycklama à Nijeholt and Jelis Boiten, MC Haaglanden, the Hague, the Netherlands. Patrick A. Brouwer and Bart J. Emmer, Erasmus MC-University Medical Center Rotterdam, the Netherlands. Sebastiaan F. de Bruijn and Lukas C. van Dijk, HAGA Hospital, the Hague, the Netherlands. L. Jaap Kappelle, University Medical Center Utrecht, the Netherlands. Rob H. Lo, University Medical Center Utrecht, the Netherlands. Ewoud J. van Dijk and Joost de Vries, Radboud University Medical Center, Nijmegen, the Netherlands. Paul L.M. de Kort and Willem Jan J. van Rooij, Sint Elisabeth Hospital, Tilburg, the Netherlands. Jan S.P. van den Berg and Boudewijn A.A.M. van Hasselt, Isala Klinieken, Zwolle, the Netherlands. Leo A.M. Aerden and René J. Dallinga, Reinier de Graaf Gasthuis, Delft, the Netherlands. Marieke C. Visser and Joseph C.J. Bot, Amsterdam UMC, location VU, Amsterdam, the Netherlands. Patrick C. Vroomen and Omid Eshghi, University Medical Center Groningen, the Netherlands. Tobien H.C.M.L. Schreuder and Roel J.J. Heijboer, Atrium Medical Center, Heerlen, the Netherlands. Koos Keizer and Alexander V. Tielbeek, Catharina Hospital, Eindhoven, the Netherlands. Heleen M. den Hertog and Dick G. Gerrits, Medical Spectrum Twente, Enschede, the Netherlands. Renske M. van den Berg-Vos and Giorgos B. Karas, Sint Lucas Andreas Hospital, Amsterdam, the Netherlands. Ewout W. Steyerberg, Erasmus MC-University Medical Center Rotterdam, the Netherlands. H. Zwenneke Flach, Isala Klinieken, Zwolle, the Netherlands. Henk A. Marquering and Marieke E.S. Sprengers, Amsterdam UMC, location AMC, the Netherlands. Sjoerd F.M. Jenniskens, Radboud University Medical Center, Nijmegen, the Netherlands. Ludo F.M. Beenen and René van den Berg, Amsterdam UMC, location AMC, the Netherlands. Peter J. Koudstaal and Wim H. van Zwam, Erasmus MC-University Medical Center Rotterdam, the Netherlands. Yvo B.W.E.M. Roos, Amsterdam UMC, location AMC, the Netherlands. Aad van der Lugt, Erasmus MC-University Medical Center Rotterdam, the Netherlands. Robert J. van Oostenbrugge, Maastricht University Medical Center and Cardiovascular Research Institute Maastricht (CARIM), the Netherlands. Charles B.L.M. Majoie, Amsterdam UMC, location AMC, the Netherlands. Diederik W.J. Dippel, Erasmus MC-University Medical Center Rotterdam, the Netherlands. van Zwam, Roos, van der Lugt, van Oostenbrugge, Majoie and Dippel contributed equally.
